# VMA21 deficiency leads to autophagic dysregulation and altered vesicle trafficking in X-linked myopathy with excessive autophagy

**DOI:** 10.1007/s00401-026-03044-z

**Published:** 2026-06-26

**Authors:** Christian A. Suarez, Sara K. Pittman, Michio Inoue, Eileen M. Lynch, Andrew Moran, Angèle N. Merlet, Emmanuelle Lacenne, Teresinha Evangelista, Conrad C. Weihl

**Affiliations:** 1 Department of Neurology, Washington University School of Medicine, St. Louis, MO 63110 USA; 2 Department of Clinical Physiology and Exercice, Myology Unit, Reference Center for Neuromuscular Diseases, Euro-NMD, University Hospital of Saint-Etienne, Saint-Priest-en-Jarez, France; 3 Interuniversity Laboratory of Human Movement Biology, Jean-Monnet University, Saint-Etienne, France; 4 Functional Unit of Neuromuscular Pathology, Department of Neuropathology, Myology Institute, Pitié-Salpêtrière Hospital, Assistance Publique Des Hôpitaux de Paris, Sorbonne University, Paris, France; 5 Department of Neurology, Washington University School of Medicine, WashU Jeffrey T. Fort Neuroscience Research Building, Room 8170, 4370 Duncan Avenue, St. Louis, MO 63110 USA

**Keywords:** XMEA, VMA21, Autophagy, Vesicle trafficking, Membrane attack complex, Vacuolar myopathy

## Abstract

X-Linked myopathy with excessive autophagy (XMEA) is a rare vacuolar myopathy caused by mutations in *Vma21,* an assembly chaperone required for vacuolar H⁺–ATPase (V-ATPase) function. However, the mechanisms linking Vma21 deficiency to progressive muscle pathology remain poorly understood, in part due to the lack of suitable animal models. To address this gap, we generated conditional *Vma21* knockout mouse models to investigate the consequences of Vma21 loss in striated muscle. Combined deletion of *Vma21* in skeletal and cardiac muscle resulted in early lethality driven by severe cardiomyopathy associated with autophagic dysregulation, preceding the development of skeletal muscle pathology. In contrast, inducible skeletal muscle-specific deletion of *Vma21* produced progressive muscle weakness and myopathy characterized by centralized nuclei, fiber splitting, and increased fiber size variability. Affected skeletal muscle also recapitulated defining pathological hallmarks of XMEA, including basal lamina reduplication and autophagic vacuoles with sarcolemmal features (AVSFs). Ultrastructural analysis revealed membrane-bound vacuoles containing partially undegraded material that frequently accumulated at the subsarcolemmal region, together with clusters of vesicular structures. Notably, mutant muscle exhibited increased staining for the late endosomal/exosomal marker CD63, which strongly colocalized with the complement membrane attack complex C5b-9. A similar increase in CD63 staining and its colocalization with C5b-9 were observed in skeletal muscle biopsies from patients with XMEA. Together, these models faithfully recapitulate key pathological features of XMEA and identify the accumulation of CD63-positive structures and their colocalization with C5b-9 as previously unrecognized features of Vma21-deficient skeletal muscle, implicating altered vesicle trafficking in XMEA pathogenesis.

**Supplementary Information:**

The online version contains supplementary material available at 10.1007/s00401-026-03044-z.

## Introduction

Intracellular pH regulation is essential for cellular homeostasis, governing processes that include protein degradation and vesicle trafficking [1]. Lysosomes require a highly acidic luminal environment to activate hydrolytic enzymes and degrade cargo [2]. Accordingly, impaired lysosomal acidification underlies a spectrum of human diseases linked to defective autophagy, including neuromuscular disorders collectively referred to as autophagic vacuolar myopathies (AVMs) [3]. A distinct subset of AVMs is defined by the presence of autophagic vacuoles with sarcolemmal features (AVSFs), vacuolar structures delineated by membranes containing sarcolemmal and basal lamina components, and includes disorders, such as Danon disease and X-linked myopathy with excessive autophagy (XMEA) [4, 5].

XMEA is a rare X-linked neuromuscular disorder affecting males, with no reported affected females to date [6]. Disease onset most commonly occurs in childhood, although congenital and late-onset presentations have been reported [3, 7–9]. XMEA is characterized by slowly progressive proximal muscle weakness, initially affecting the lower limbs and later involving additional muscle groups, often leading to loss of ambulation by mid-adulthood [10]. Although XMEA has traditionally been considered a skeletal muscle-specific disorder, increasing clinical evidence indicates that respiratory involvement is relatively common, and cardiac abnormalities have been reported in a subset of patients [10, 11].

XMEA is caused by mutations in *Vma21,* which encodes an essential assembly chaperone for the vacuolar H⁺–ATPase (V-ATPase), the primary proton pump responsible for lysosomal acidification in eukaryotes [12]. Loss of Vma21 disrupts V-ATPase assembly, resulting in defective lysosomal degradation and accumulation of autophagic vacuoles [6, 10]. Histopathological analysis of skeletal muscle from XMEA patients reveals cytoplasmic vacuoles that stain positively for sarcolemmal and basal lamina proteins, complex fiber splitting, deposition of complement membrane attack complex (C5b-9), and, in some cases, aggregates of autophagy receptors such as SQSTM1/p62 [8, 10]. Robust positivity of the lysosomal marker LAMP2 distinguishes XMEA from Danon disease, which is caused by loss-of-function mutations in the *LAMP2* gene [8]. Ultrastructural analyses further demonstrate clustered lysosomes and vacuoles located in subsarcolemmal and intermyofibrillar regions, along with reduplication of the basal lamina [8, 10]. In addition, autophagic material has been observed between layers of the basal lamina and within the extracellular matrix, suggestive of exocytosis of autophagic material in XMEA patient muscle [10].

Despite identification of *Vma21* as the genetic cause of XMEA, how Vma21 deficiency leads to progressive muscle pathology in vivo remains incompletely understood, and suitable mammalian models that faithfully recapitulate the defining structural features of XMEA have been limited. Here, we generated and characterized mouse models of Vma21 deficiency to establish an in vivo system that recapitulates the pathological features of XMEA. We show that skeletal muscle-specific *Vma21* deletion recapitulates hallmark pathological features of XMEA, including progressive myopathy, autophagic dysregulation and formation of AVSFs, while combined skeletal and cardiac muscle deletion results in early lethal cardiomyopathy with autophagic impairment. These models establish a physiologically relevant in vivo system for studying XMEA pathogenesis and evaluating disease-modifying therapeutic strategies.

## Materials and methods

### Generation of Vma21 conditional knockout mice strains

Vma21 conditional knockout mice were generated using the CRISPR-Cas9 system to introduce 2 loxP sites, flanking upstream of promoter and last intron, into the same allele of the X-linked *Vma21* gene. We designed gRNAs targeting upstream from the *Vma21* promoter and its last intron and oligonucleotides, containing loxP sites and BamHI or HindIII restriction sites, flanked by 60 bp homologous to the targeted regions. Zygotes were microinjected with a mixture of Cas9 mRNA, gRNAs, and oligos and transferred to pseudopregnant female mice. Genotyping of 8 F0 born pups using tail-extracted DNA and Vma21-specific primer sets flanking the loxP sites led to the identification of one female founder mouse carrying the loxP sites on both alleles of the X chromosomes. The founder mice were healthy, normal in size, and did not display any phenotype compared to their wild-type littermates.

### Animals and experimental protocols

Control [(C57BL/6, Stock No: 000664), ACTA1p-cre/Esr1 (Tg(ACTA1-cre /Esr1*)2Kesr/J, Stock No: 025750 HSA-MCM), and B6.FVB(129S4)-Tg(Ckmm-cre)5Khn/J, Stock No: 006475] mice were purchased from Jackson Laboratories. All animal experimental protocols were approved by the Animal Studies Committee of Washington University School of Medicine per IACUC guidelines. Mice were housed in a temperature-controlled environment with 12-h light–dark cycles where they received food and water ad libitum. Tamoxifen-supplemented water was prepared by dissolving 1 g tamoxifen citrate (Goldbio; T-750–2) into 2L of DI water.

### Genotyping of mice

Genomic DNA was extracted from mutants and control mice tails using KAPA Express DNA extraction Kit (Kapabiosystems, KK7103) and standard PCR was performed using Lambda Biotech (206,811) and gene-specific primers. PCR products were subjected to electrophoresis on a 2.5% agarose gel. After traditional PCR verification of germline transmission of X-linked floxed alleles in F1 litters, mouse genotypes were determined by real-time PCR analysis (Transnetyx, Cordova, TN, USA) using tail biopsy samples.

### Histochemistry/immunofluorescence

For histological analysis of hearts, mice were transcardially perfused with phosphate-buffered saline (PBS; Gibco, 14,190–136). Hearts were dissected and placed individually into scintillation vials containing sufficient 3.7% paraformaldehyde (PFA) to fully submerge the tissue, and immersion-fixed overnight at 4 °C. Following fixation, hearts were rinsed three times in PBS and cryoprotected by immersion in 30% (w/v) sucrose in PBS overnight at 4 °C. Tissues were then embedded in optimal cutting temperature (OCT) compound, frozen on dry ice, and stored at − 80 °C until ready to be sectioned to a 10 μm thickness.

For histological analysis of skeletal muscle, samples were mounted in tragacanth gum (10% solution, Sigma-Aldrich, G1128), flash-frozen in 2-methylbutane over liquid nitrogen, and stored at − 80 °C until ready to be sectioned to an 8 μm thickness.

For the H&E stain, a 1% aqueous solution of eosin Y (Sigma E-6003) was prepared in deionized water, and Harris hematoxylin stain (Lerner Laboratories, 1,931,382) was filtered before use. Slides in a metal staining rack were immersed in the filtered Harris hematoxylin for 10 s, then transferred to a beaker of tap water, and rinsed until the water was clear. Then, the slides were immersed in eosin stain for 30 s and again rinsed with tap water. Then, sections were dehydrated in ascending alcohol solutions (50%, 70%, 80%, 95% × 2, 100% × 2) and cleared with xylene three or four times, and a glass coverslip was mounted to the glass slide using Permount.

For esterase enzyme histochemistry, cryosections of snap-frozen skeletal muscle (8 μm) were incubated at room temperature in a staining solution containing α-naphthyl acetate and pararosaniline-based coupling reagents prepared fresh prior to use, as previously described. Sections were then rinsed in water, dehydrated through graded alcohols, cleared in xylene, and mounted with Permount.

For acid phosphatase enzyme histochemistry, Sects. (8 μm) were incubated in a naphthol phosphate-based substrate solution containing diazonium coupling reagents prepared fresh prior to use, as described in standard protocols. Following incubation, sections were washed, dehydrated through graded alcohols, cleared in xylene, and mounted with Permount.

For immunostaining, sections were fixed using 3.7% PFA for 10 min followed by 10 min of ice-cold acetone. The muscle sections were then permeabilized for 10 min in 0.5% Triton X-100 and blocked for 1 h at room temperature in PerkinElmer blocking reagent (FP1012). Primary antibodies were diluted in blocking reagent and incubated at 4 °C overnight. After three rinses for 5 min each with 1 × PBS, secondary antibodies were added to the slides at 1:500 dilution in blocking reagent and incubated for 1 h at room temperature. Slides were rinsed with 1 × PBS again three times for 5 min each and then incubated for 10 min with 4′,6-diamidino-2-phenylindole (DAPI; 1 μg/ml) followed by a final three PBS rinses. A cover glass was mounted to slides using Mowiol 4–88 (Sigma-Aldrich, 81,381). The following antibodies and dilutions were used for immunostaining: rabbit anti-laminin (abcam; ab11575; 1:500), rabbit anti-Caveolin-3 (Thermo Fisher Scientific; PA1-066; 1:250); mouse anti-dystrophin (Millipore Sigma; D8043; 1:250), rat anti-LAMP2 (abcam; ab13524; 1:200), rabbit anti-LC3B (Millipore Sigma; L7543; 1:200), rabbit anti-SQSTM1 (Proteintech; 18,420–1-AP; 1:1000), mouse anti-Ubiquitin (P4D1) (Cell Signaling; 3936; 1:200), and mouse anti-C5b-9 (Dako; M0777; 1:200).

Frozen skeletal muscle Sects. (8 μm) from patients with genetically confirmed *Vma21* mutations and from one control individual were analyzed. Sections were fixed in cold acetone (2 × 10 min) and incubated overnight at 4 °C with primary antibodies against C5b-9 (mouse monoclonal IgG2a, Dako M0777; 1:50) and CD63 (mouse monoclonal IgG1, Novus Biologicals NBP2-42,225; 1:250). After washing in phosphate-buffered saline (PBS), sections were incubated for 1 h at room temperature with isotype-specific secondary antibodies: Alexa Fluor 488 goat anti-mouse IgG2a (Invitrogen A21131; 1:250) and Alexa Fluor 568 goat anti-mouse IgG1 (Invitrogen A21124; 1:250). Nuclei were counterstained with DAPI, and sections were mounted in Mowiol.

### Imaging and image analysis

Immunofluorescently labeled cardiac and skeletal muscle sections were imaged using a Nikon CSU-W1 spinning disk confocal microscope (Nikon Instruments, Melville, NY). Images were acquired using NIS-Elements HC software (Nikon Instruments). Human skeletal muscle sections were imaged using a Zeiss Axioplan 2 microscope equipped with an Axiocam 305 mono camera. Image acquisition settings were kept identical across samples within each experiment. Hematoxylin and eosin (H&E)-stained mouse heart and skeletal muscle sections were imaged using a Leica DM4 B microscope (Leica Microsystems, Germany), and images were acquired using Leica Application Suite X (LAS X) software.

For histological quantification, three non-overlapping fields per sample were acquired at 40 × objective (400 × total magnification). Percentage of fibers with internal nuclei, percentage of fibers exhibiting splitting, and fiber size variability (percent coefficient of variation) were quantified using Fiji (ImageJ, National Institutes of Health).

### Transmission electron microscopy

Tibialis anterior, gastrocnemius, and quadriceps muscles were dissected from control and HSA-*Vma21*KO mice after 4 months of tamoxifen treatment and fixed overnight at 4 °C in fixative solution (2.5% glutaraldehyde, 2% PFA, 0.15 M cacodylate buffer, pH 7.4, with 2 mM CaCl_2_). After fixation, samples were rinsed in 0.15 M cacodylate buffer containing 2 mM calcium chloride three times for 10 min each followed by a secondary fixation in 1% osmium tetroxide and 1.5% potassium ferrocyanide in 0.15 M cacodylate buffer containing 2 mM calcium chloride for 1 h in the dark. The samples were then rinsed three times for 10 min each in ultrapure water and en bloc stained with 2% aqueous uranyl acetate overnight at 4 °C in the dark. After four washes for 10 min each in ultrapure water, the samples were dehydrated in a graded acetone series (10%, 30%, 50%, 70%, 90%, 100% × 3) for 10 min each step, infiltrated with Spurr’s resin (Electron Microscopy Sciences), and embedded and polymerized at 60 °C for 48 h. After curing, 70-nm-thin sections were cut and imaged on a TEM (JEOL JEM-1400Plus) at 120 kV.

### Western blotting

Heart or muscle tissue lysates were collected in RIPA buffer with protease inhibitor cocktail. Protein concentrations were obtained through BCA assay and measured with the BioTek Epoch plate reader at 562 nm. Each lane of a SDS-PAGE gel was loaded with 20 to 30 μg of total protein, and gels were run at 80 V for 2 h and 20 min. Next, the samples were transferred to PVDF membranes at 0.1 mA per rig overnight. Membranes were blocked with 5% milk for 1 h, and then primary antibodies were added and incubated overnight at 4 °C. Blots were treated with horseradish peroxidase secondary antibodies at a 1:5000 dilution in 5% milk for 1 h at room temperature and then rinsed three times with 1 × PBS-Tween before imaging membranes. Membranes were imaged using enhanced chemiluminescence (Cytiva Amersham, RPN2209) and a Syngene G:Box Chemi XT4 imager. The following antibodies and dilutions were used for western blotting: rabbit anti-VMA21 (abcam; ab242115; 1:250), rat anti-LAMP2 (abcam; ab13524; 1:500), rabbit anti-LC3B (Millipore Sigma; L7543; 1:500), mouse anti-SQSTM1 (Novus Biologicals; H00008878-M01; 1:1000), mouse anti-Ubiquitin (P4D1) (Cell Signaling; 3936; 1:1000), and rabbit anti-GAPDH (Cell Signaling; 2118; 1:1000). The following gel percentages were used: 15% gels for LC3B and VMA21; 10% gels for SQSTM1, Ubiquitin (P4D1), and LAMP2.

### Inverted screen (mesh) test

Muscle strength and motor coordination were assessed using the inverted screen test. The apparatus consisted of a wire mesh grid (12 mm × 12 mm squares made of 1 mm diameter wire) mounted within a PVC frame (52 cm × 21.5 cm). Mice were placed in the center of the mesh and allowed to grip the grid with all four limbs. The screen was then inverted 180° and held approximately 40–50 cm above a padded surface. The latency to fall was recorded, with a maximum cutoff time of 60 s. Each mouse was tested in two trials separated by a 2–5 min rest period, and the average latency to fall was used for analysis.

### Transthoracic echocardiography

Non-invasive ultrasound examination of the cardiovascular system was performed using a Vevo Ultrasound System (VisualSonics Inc, Toronto, Ontario, Canada) according to the following procedures. First, mice were lightly anesthetized with an intraperitoneal injection of 2% Avertin (tribromoethanol, 0.005 ml/g). Hair was removed from the anterior chest with a combination of shaving and chemical hair remover, and the animals were placed on a warming pad in a left lateral decubitus position to maintain normothermia. Ultrasound coupling gel was applied to the chest. Cursory examination of cardiac structure and function under physiologic conditions was obtained with hand-held manipulation of the ultrasound transducer. Complete two-dimensional, M mode, and Doppler ultrasound examination was performed from multiple views.

### Statistical analysis

All quantitative data were analyzed using GraphPad Prism (GraphPad Software). Data are presented as mean unless otherwise indicated. Comparisons between two groups were performed using an unpaired two-tailed Student’s *t*-test. Experiments involving two independent variables were analyzed using two-way analysis of variance (ANOVA) followed by Šidák’s multiple comparisons test. Survival analysis was performed using Kaplan–Meier survival curves, and statistical significance between groups was assessed using log-rank (Mantel–Cox) test. A *p* value < 0.05 was considered statistically significant. In all cases, *P < 0.05, **P < 0.01, and ***P < 0.001.

## Results

### Deletion of *Vma21* in striated muscle results in early postnatal lethality

**Fig. 1:**
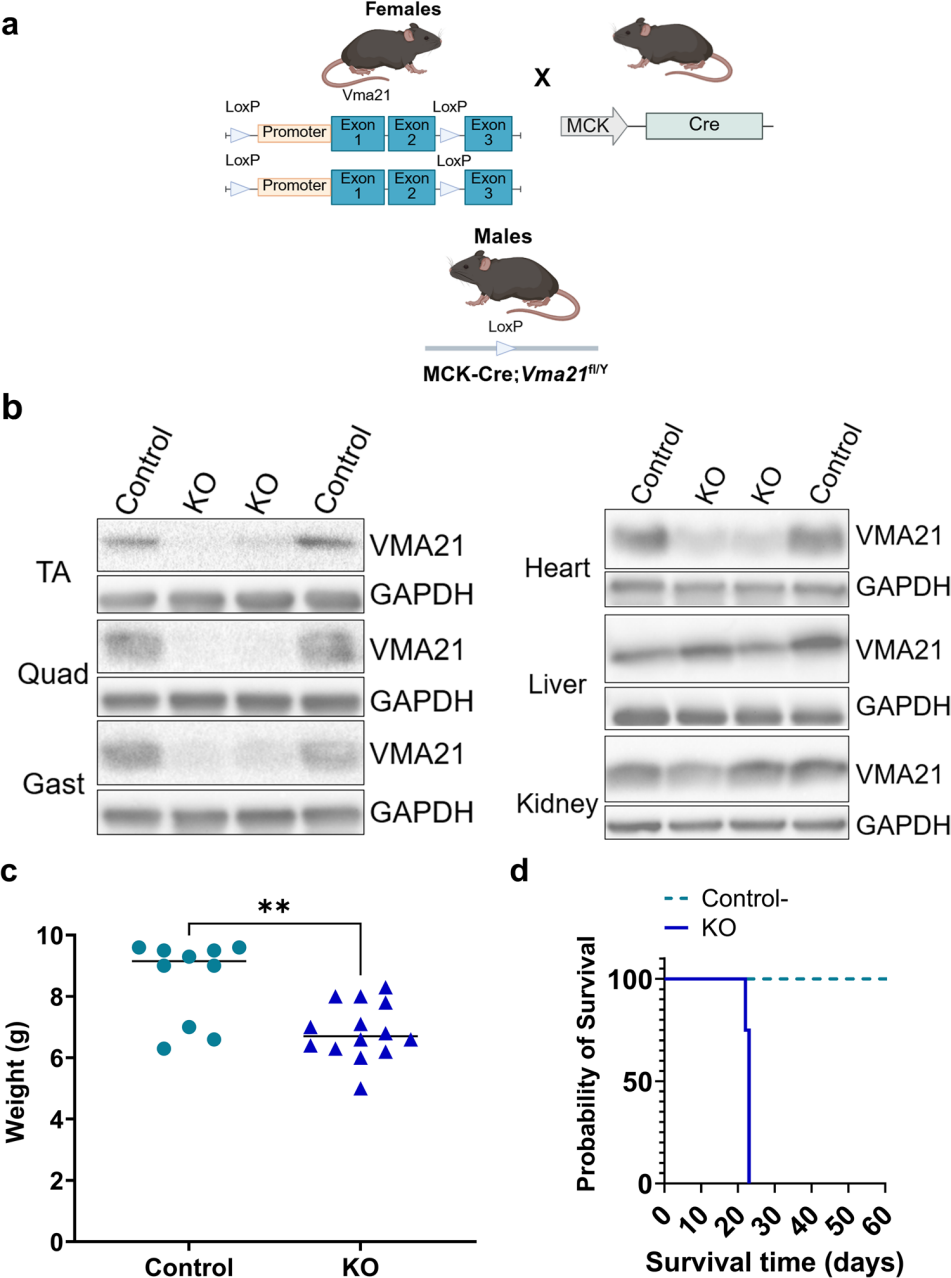
Striated muscle-specific deletion of *Vma21* results in reduced body weight and shortened lifespan. **a** Breeding strategy used to generate MCK-Cre;*Vma21*^fl/Y^ mice. **b** Immunoblot analyses of lysates from tibialis anterior (TA), quadriceps (Quad), gastrocnemius (Gast), heart, liver, and kidney of P20 *Vma21*^fl/Y^ (control) or MCK-Cre;*Vma21*^fl/Y^ (KO) mice probed with antibodies against Vma21 or GAPDH. **c** Body weight of control and KO mice at P20. **d** Kaplan–Meier survival curves of control and KO mice. *n* = 7 control and n = 8 KO mice. Comparison between groups was performed using an unpaired two-tailed Student’s *t*-test. Survival curves were compared using a log-rank (Mantel–Cox) test. *p < 0.05; **p < 0.01; ***p < 0.001; n.s., not significant

To investigate the consequences of striated muscle-specific loss of Vma21 in vivo, we developed a conditional *Vma21* gene inactivation strategy utilizing the Cre-LoxP system. *Vma21*^fl/fl^ mice were generated by inserting a LoxP site approximately 18 kb upstream of the *Vma21* promoter and a second LoxP site within the final intron of the gene. Recombination between these sites results in deletion of the entire *Vma21* transcript. *Vma21*^fl/fl^ mice were then crossed with MCK-Cre transgenic mice, in which Cre recombinase is expressed under control of the muscle creatine kinase (MCK) promoter, driving expression in differentiated skeletal and cardiac muscle (Fig. 1a). Because *Vma21* is located on the X chromosome, male mice were used to ensure complete gene inactivation.

MCK-Cre;*Vma21*^fl/Y^ mice (hereafter referred to as MCK-*Vma21*KO) were compared with Cre-negative *Vma21*^fl/Y^ littermates, which served as controls. Efficient loss of Vma21 protein in both skeletal and cardiac muscle of MCK-*Vma21*KO mice was confirmed by immunoblotting at postnatal day 20 (P20) (Fig. 1b). MCK-*Vma21*KO mice exhibited significantly reduced body weight by P20 compared with littermate controls (Fig. 1c). Strikingly, MCK-*Vma21*KO mice displayed a dramatically shortened lifespan, with 100% mortality occurring between postnatal days 22 and 23 (Fig. 1d).

### Deletion of *Vma21* causes severe cardiomyopathy with autophagic dysregulation

**Fig. 2:**
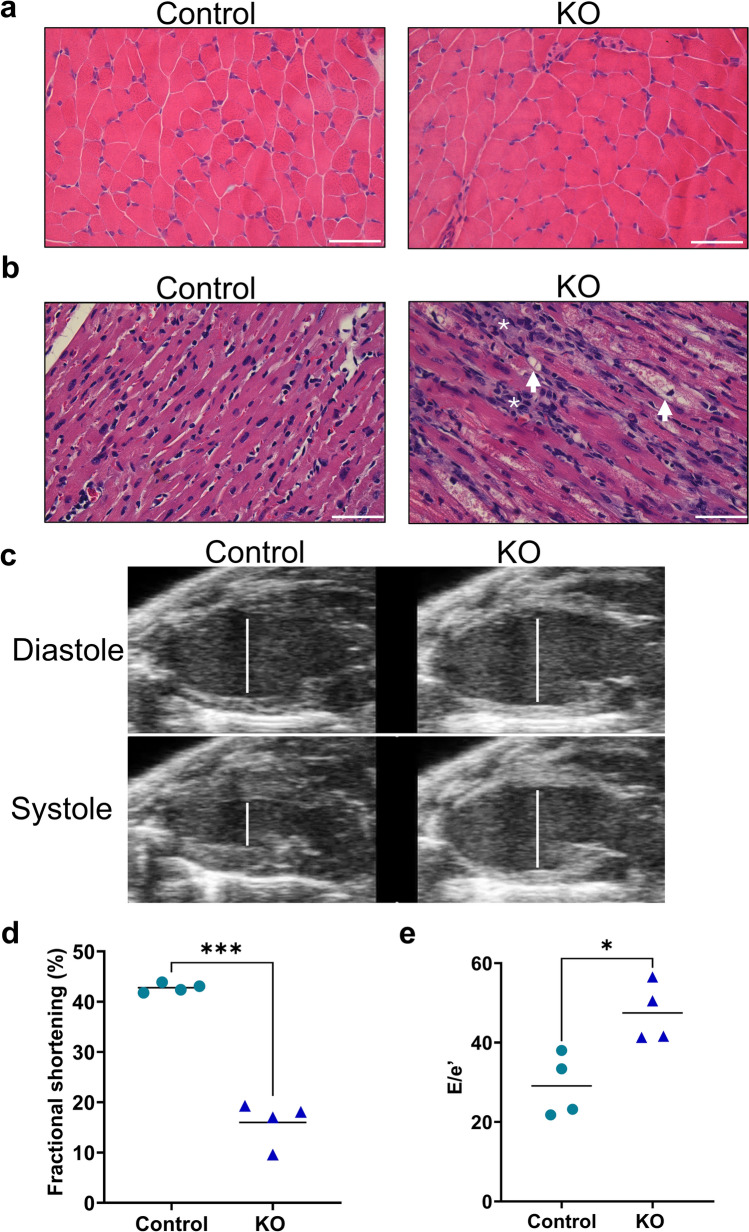
Deletion of *Vma21* in the heart leads to cardiomyopathy. **a** Hematoxylin and eosin (H&E) staining of gastrocnemius muscle from P20 Control or KO mice. **b** H&E staining of hearts from P20 *Vma21*^fl/Y^ (Control) or MCK-Cre;*Vma21*^fl/Y^ (KO) mice. White arrows indicate vacuolated cardiomyocytes. Asterisks indicate inflammatory infiltrates. **c** Representative echocardiographic images of P20 Control and KO mice at diastole (top) and systole (bottom). Vertical white lines indicate ventricular internal diameter. Quantification of fractional shortening (**d**) and E/e′ ratio (**e**) from echocardiographic analysis. Comparison between groups was performed using an an unpaired two-tailed Student’s *t*-test. *p < 0.05; **p < 0.01; ***p < 0.001; n.s., not significant. Scale bars: 50 μm

To determine whether the loss of Vma21 in skeletal muscle leads to myopathy that could explain the observed survival phenotype, hematoxylin and eosin (H&E) staining was performed on cryosections of skeletal muscle from control and MCK-*Vma21*KO mice at P20. Despite efficient *Vma21* deletion, MCK-*Vma21*KO skeletal muscle did not exhibit evident markers of myopathy, such as internalized nuclei or increased fiber size variability, at this time point (Fig. 2a). However, given that *Vma21* was also efficiently deleted in cardiac tissue, we performed a histopathological evaluation of the heart. In contrast to the skeletal muscle, MCK-*Vma21*KO hearts at P20 revealed hallmarks of severe cardiomyopathy, characterized by inflammatory infiltration, and extensive cytoplasmic vacuolization (Fig. 2b). Functional assessment by transthoracic echocardiography at P18 demonstrated markedly impaired contractile function in MCK-*Vma21*KO mice (Fig. 2c). Quantitative analysis revealed significantly reduced fractional shortening, an index of left ventricular systolic function (Fig. 2d), and increased E/e′, an index of left ventricular filling pressure (Fig. 2e) compared with controls, identifying heart failure as the most likely cause of lethality.

**Fig. 3:**
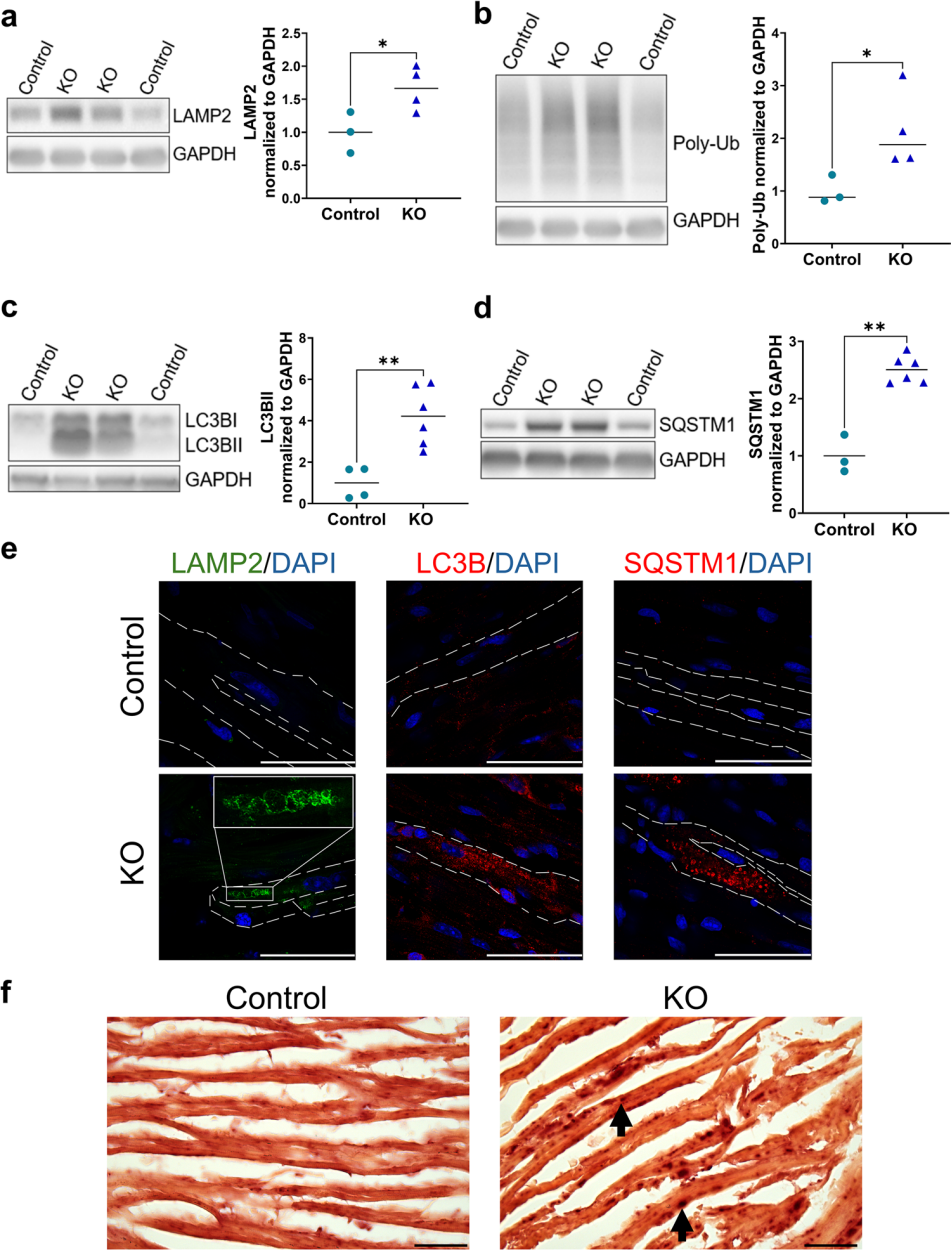
Deletion of *Vma21* in cardiac muscle leads to autophagic dysregulation. Immunoblot analyses of heart lysates from P20 *Vma21*^fl/Y^ (control) and MCK-Cre;*Vma21*^fl/Y^ (KO) mice using antibodies against LAMP2 (**a**), poly-ubiquitinated proteins (P4D1) (**b**), LC3B (**c**), SQSTM1 (**d**), or GAPDH and corresponding quantifications. **e** Immunofluorescence staining for LAMP2 (green), LC3B (red), or SQSTM1 (red) on heart cryosections from P20 control and KO mice. DAPI (blue) stains nuclei. Dashed lines outline cardiomyocyte boundaries. Inset shows higher-magnification view of the indicated region. **f** Acid phosphatase enzyme histochemistry on heart sections from P20 control and KO mice. Black arrows indicate acid phosphatase granules. Comparison between groups was performed using an unpaired two-tailed Student’s *t*-test. *p < 0.05; **p < 0.01; ***p < 0.001; n.s., not significant. Scale bars: 50 μm. LAMP2 and poly-ubiquitin immunoblots were generated from the same heart lysates and run in parallel gels. GAPDH was probed on the LAMP2 membrane and used as the loading control for both panels

Given the observed vacuolization and the established role of Vma21 in lysosomal acidification (6), we examined whether the observed cardiomyopathy was associated with impaired autophagy. Immunoblot analysis of cardiac tissue revealed increased levels of LAMP2, poly-ubiquitinated proteins, LC3B-II, and SQSTM1 (Figs.). Immunofluorescence analysis further demonstrated accumulation of LAMP2 positive structures, increased LC3B- and SQSTM1 positive puncta (Fig. 3e). Acid phosphatase enzyme histochemistry revealed prominent staining within vacuolar structures in cardiomyocytes of MCK-*Vma21*KO hearts (Fig. 3f). In contrast, immunoblot analysis of skeletal muscle (tibialis anterior, gastrocnemius, and quadriceps) at P20 revealed no significant differences in LC3B-II or SQSTM1 levels between controls and MCK-*Vma21*KO mice (Fig. S1). Together, these findings indicate a pathological phenotype consistent with impaired autophagy in Vma21-deficient hearts, while no significant changes in bulk autophagy markers are observed in skeletal muscle at this early stage.

### Generation of a skeletal muscle-specific inducible *Vma21* knockout model

**Fig. 4:**
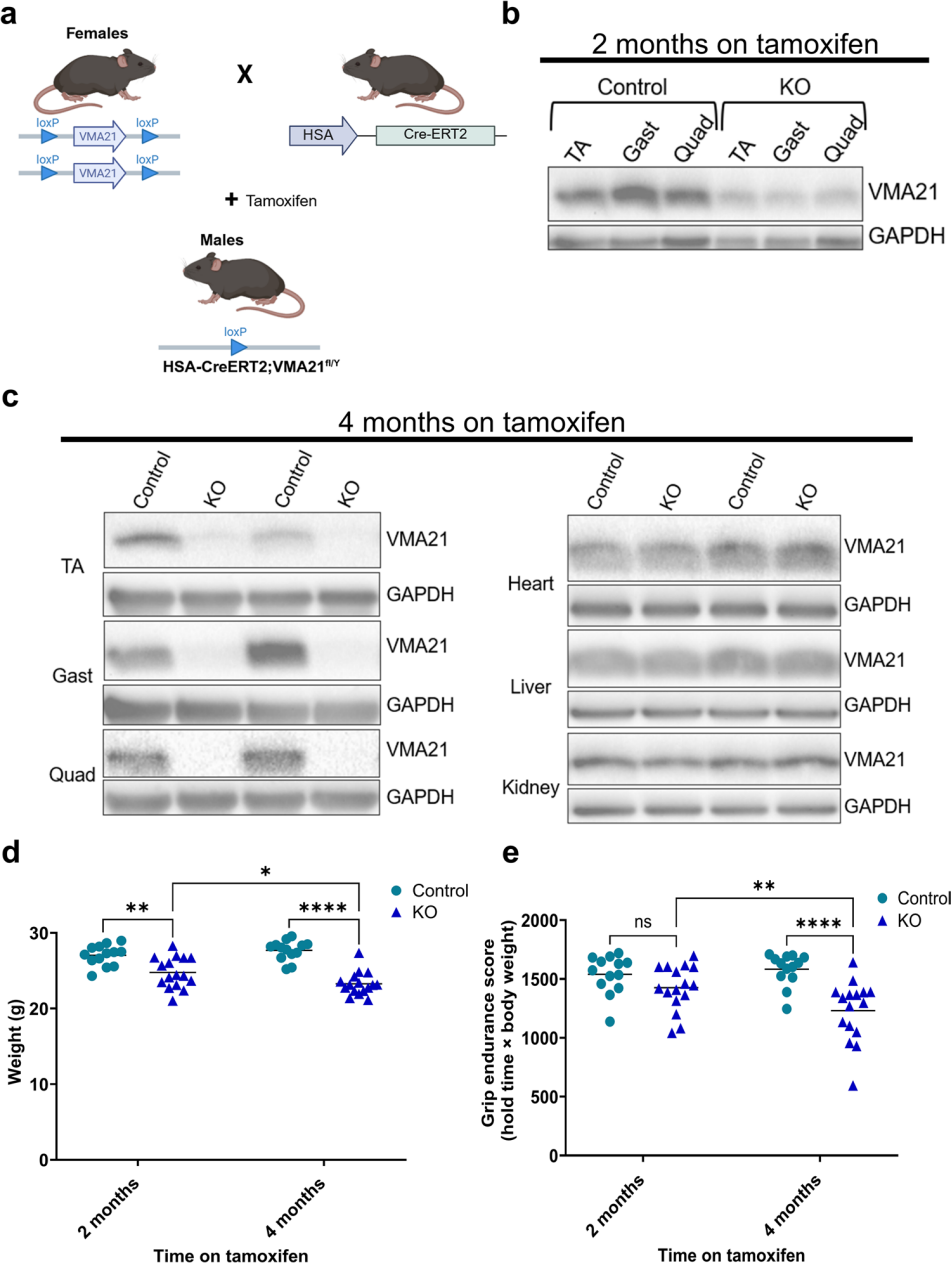
Skeletal muscle-specific deletion of *Vma21* results in reduced body weight and progressive muscle weakness. **a** Breeding strategy used to generate HSA-CreERT2;*Vma21*^fl/Y^ mice. **b** Immunoblot analyses of lysates from tibialis anterior (TA), quadriceps (Quad), and gastrocnemius (Gast) muscles from *Vma21*^fl/Y^ (control) or HSA-CreERT2;*Vma21*^fl/Y^ (KO) mice after 2 months of tamoxifen treatment, probed with antibodies against Vma21 or GAPDH. **c** Immunoblot analysis of lysates from TA, Quad, Gast, heart, liver, and kidney from control or KO mice after 4 months of tamoxifen treatment, probed with antibodies against Vma21 or GAPDH. **d** Body weight of control and KO mice after 2 and 4 months of tamoxifen treatment. **e** Grip endurance score (hold time × body weight) of control and KO mice after 2 and 4 months of tamoxifen treatment. Comparison between groups was performed using an unpaired two-tailed Student’s *t*-test. *p < 0.05; **p < 0.01; ***p < 0.001; n.s., not significant

Because X-linked myopathy with excessive autophagy (XMEA) primarily affects skeletal muscle, we sought to determine the consequences of *Vma21* deletion specifically in skeletal muscle while avoiding the early cardiomyopathy and lethality observed in the MCK-Cre model. To achieve this, we generated a tamoxifen-inducible HSA-CreERT2; *Vma21*^fl/Y^ mouse line, in which Cre recombinase expression is restricted to skeletal muscle upon tamoxifen treatment **(**Fig. 4a**)**. HSA-CreERT2;*Vma21*^fl/Y^ mice (hereafter referred to as HSA-*Vma21*KO) were compared with Cre-negative *Vma21*^fl/Y^ littermates, which served as controls. This strategy prevents cardiac involvement and enables temporal control of *Vma21* deletion in adult mice, thereby bypassing embryonic or early postnatal lethality.

Tamoxifen was administered ad libitum through drinking water to induce Cre-mediated recombination, and both HSA-*Vma21*KO mice and control littermates were treated with tamoxifen under identical conditions. After two months of tamoxifen treatment, immunoblot analysis revealed a robust reduction of Vma21 protein in multiple skeletal muscles, including tibialis anterior (TA), gastrocnemius (Gast), and quadriceps (Quad), in HSA-*Vma21*KO mice compared with control littermates (Fig. 4b). Vma21 protein levels remained markedly reduced in skeletal muscle after four months of tamoxifen treatment, whereas expression in non-skeletal tissues (heart, liver, and kidney) was unchanged, confirming skeletal muscle-specific and persistent reduction of Vma21 (Fig. 4c).

Because *Vma21* is located on the X chromosome, male mice were used in these experiments to ensure complete gene inactivation. Phenotypic differences between control littermates and HSA-*Vma21*KO mice became apparent following prolonged *Vma21* deletion. HSA-*Vma21*KO mice exhibited reduced body weight compared with control littermates after two months on tamoxifen treatment, and this difference became more pronounced after four months of tamoxifen treatment (Fig. 4d). Importantly, consistent with the progressive muscle weakness characteristic of XMEA, HSA-*Vma21*KO mice also displayed a significant reduction in grip endurance compared with littermate controls after four months of tamoxifen treatment (Fig. 4e).

### Skeletal muscle-specific deletion of *Vma21* leads to progressive myopathy

**Fig. 5:**
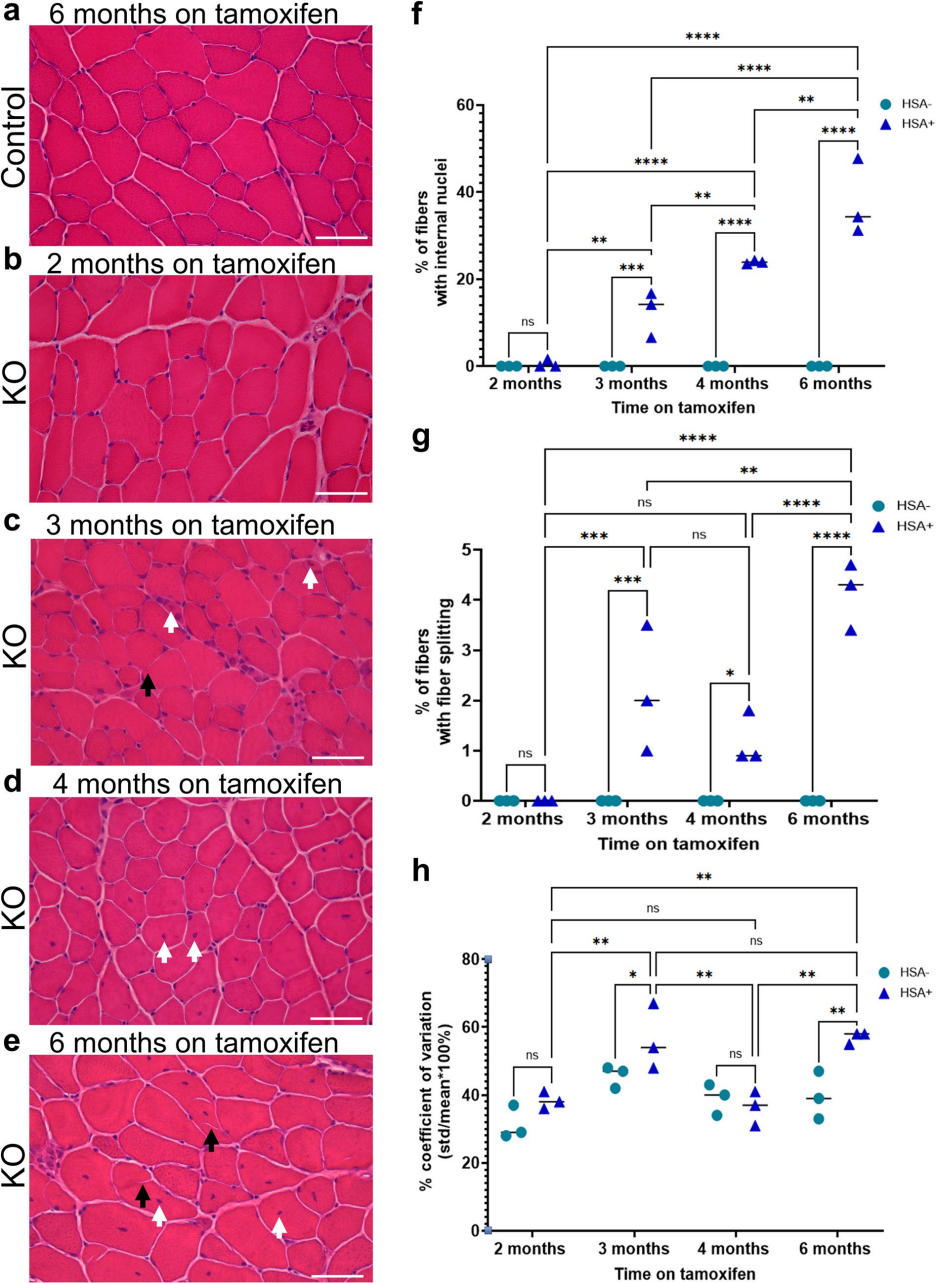
Skeletal muscle-specific deletion of Vma21 results in progressive myopathy. Hematoxylin and eosin (H&E) staining of tibialis anterior (TA) muscle from Vma21fl/Y (control) mice after 6 months of tamoxifen treatment (**a**) and from HSA-CreERT2;Vma21fl/Y (KO) mice after 2 months (**b**), 3 months (**c**), 4 months (**d**), or 6 months (**e**) of tamoxifen treatment. White arrows indicate centrally located nuclei. Black arrows indicate fiber splitting. Quantification of the percentage of fibers containing centrally located nuclei (**f**), percentage of fibers exhibiting splitting (**g**), and coefficient of variation of myofiber cross-sectional area (**h**). Each data point represents the average of three images per mouse. Data were analyzed using two-way ANOVA with multiple comparisons. *p < 0.05; **p < 0.01; ***p < 0.001; n.s., not significant. Scale bars: 50 μm

To determine whether skeletal muscle-specific deletion of *Vma21* results in myopathy, we performed histological analysis of skeletal muscle after two, three, four, and six months of tamoxifen treatment. Hematoxylin and eosin (H&E) staining revealed no apparent histopathological abnormalities in skeletal muscle from control mice after six months on tamoxifen or in HSA-*Vma21*KO skeletal muscle after two months on tamoxifen (Figs.). At three months of tamoxifen treatment, HSA-*Vma21*KO muscle exhibited increased variability in fiber size, accompanied by the appearance of fibers containing internalized nuclei and early signs of fiber splitting (Fig. 5c). By four months of tamoxifen treatment, HSA-*Vma21*KO muscle exhibited an increased number of fibers containing internal nuclei, while fiber splitting remained similar to three months and fiber size variability was reduced relative to that time point (Fig. 5d). After six months on tamoxifen, these pathological features became more pronounced, with increased fiber splitting, a greater number of fibers containing internal nuclei, and increased fiber size variability (Fig. 5e).

Quantitative analysis confirmed the progressive nature of these myopathic changes. The percentage of fibers exhibiting splitting or containing internal nuclei was significantly higher in HSA-*Vma21*KO skeletal muscle beginning at three months and further increased after six months of tamoxifen treatment (Fig. 5f, g). In addition, analysis of fiber size variability revealed a significant increase in the coefficient of variation of myofiber cross-sectional area at three months, followed by a reduction at four months and a subsequent increase after six months of tamoxifen treatment, suggesting dynamic changes in fiber size over the course of disease progression (Fig. 5h).

Together, these findings demonstrate that skeletal muscle-specific deletion of *Vma21* results in a progressive myopathy characterized by fiber splitting, internal nucleation, and increased fiber size variability, consistent with pathological features observed in XMEA.

### Skeletal muscle-specific deletion of *Vma21* results in autophagic dysregulation

**Fig. 6:**
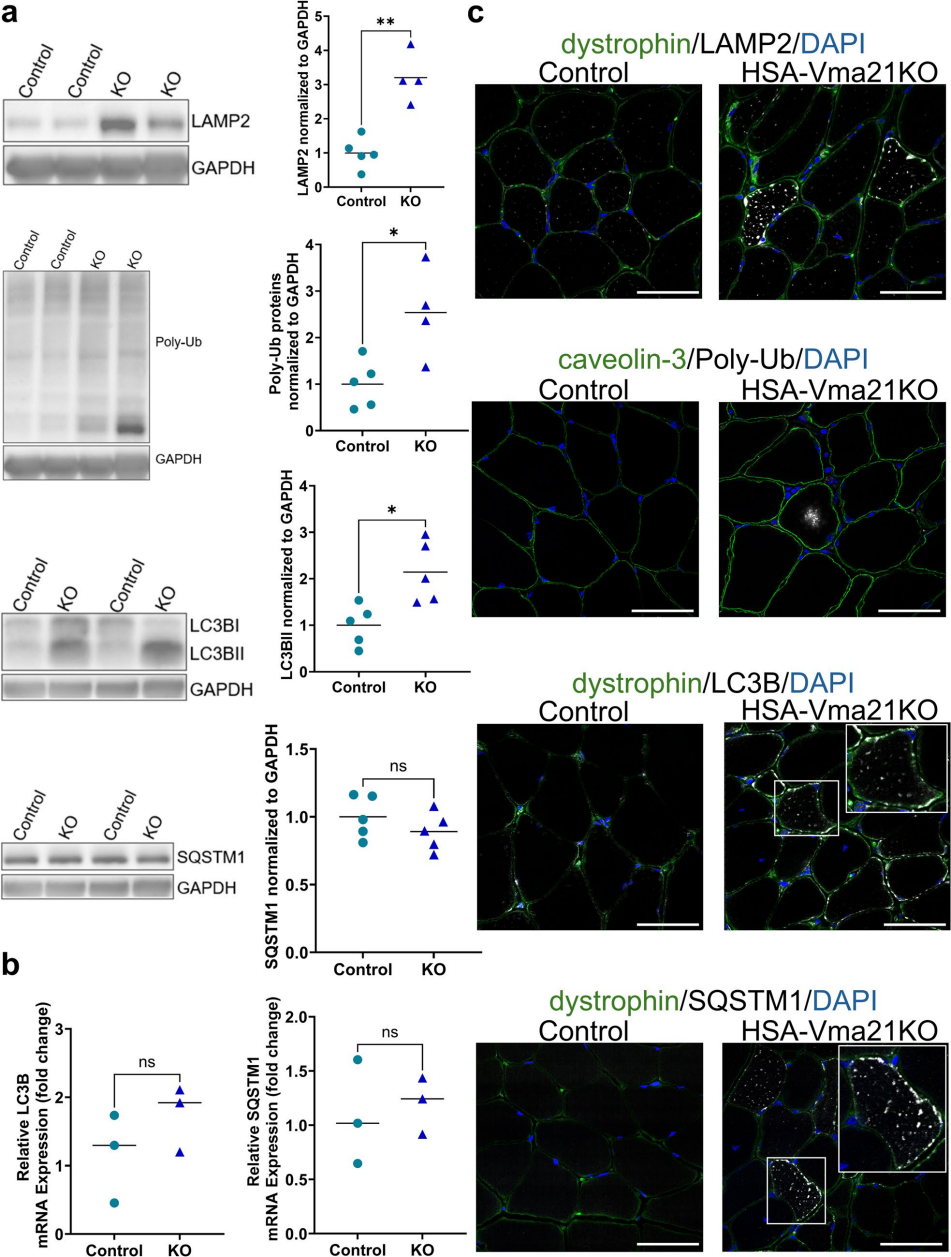
Skeletal muscle-specific deletion of *Vma21* leads to autophagic dysregulation. **a** Immunoblot analysis of gastrocnemius muscle lysates from *Vma21*^fl/Y^ (control) or HSA-CreERT2;*Vma21*^fl/Y^ (KO) mice after 4 months of tamoxifen treatment using antibodies against LAMP2, poly-ubiquitinated proteins (P4D1), LC3B, SQSTM1, or GAPDH and corresponding quantifications. **b** RT–qPCR analysis of *Lc3b* and *Sqstm1* mRNA in gastrocnemius muscle from control and KO mice after 4 months of tamoxifen treatment. **c** Immunofluorescence staining on cryosections of gastrocnemius muscle from control or KO mice after 4 months of tamoxifen treatment using antibodies against dystrophin (green) and LAMP2 (white), caveolin 3 (green) and poly-ubiquitinated proteins (P4D1) (white), dystrophin (green) and LC3B (white), or dystrophin (green) and SQSTM1 (white). DAPI (blue) stains nuclei. Insets show higher-magnification views of the indicated regions. Comparison between groups was performed using an unpaired two-tailed Student’s *t*-test. *p < 0.05; **p < 0.01; ***p < 0.001; n.s., not significant. Scale bars: 50 μm. LAMP2 and poly-ubiquitin immunoblots were generated from the same samples and run on parallel gels; GAPDH from the LAMP2 membrane is shown as the loading control for both panels. LC3B and SQSTM1 immunoblots were obtained from the same membrane and therefore share the same GAPDH loading control

Building on the progressive myopathy observed following prolonged skeletal muscle-specific deletion of *Vma21*, and given the established role of Vma21 in lysosomal function, we next examined whether loss of *Vma21* disrupts autophagy in skeletal muscle. Immunoblot analysis of gastrocnemius muscle after four months of tamoxifen treatment revealed increased levels of LAMP2, poly-ubiquitinated proteins, and LC3B-II in HSA-*Vma21*KO mice compared with control littermates (Fig. 6a). In contrast, RT-qPCR analysis showed no significant differences in *Lc3b* or *Sqstm1* mRNA expression between controls and HSA-*Vma21*KO gastrocnemius muscle (Fig. 6b), suggesting that the observed accumulation of autophagy-related proteins does not result from transcriptional upregulation. Immunofluorescence analysis further demonstrated prominent accumulation of LAMP2, poly-ubiquitin, LC3B, and SQSTM1 positive structures within HSA-*Vma21*KO muscle fibers (Fig. 6c). Notably, LAMP2, LC3B, and SQSTM1 positive puncta were frequently enriched toward the fiber periphery. Surprisingly, no increase in punctate staining for LAMP2, LC3B, SQSTM1, or poly-ubiquitinated proteins was detected in HSA-*Vma21*KO skeletal muscle after two months of tamoxifen treatment (Fig. S2).

Together, these findings indicate that skeletal muscle-specific deletion of *Vma21* results in accumulation of lysosomal and autophagy-related structures, consistent with autophagic dysregulation.

### *Vma21* deletion recapitulates key histopathological hallmarks of XMEA

**Fig. 7:**
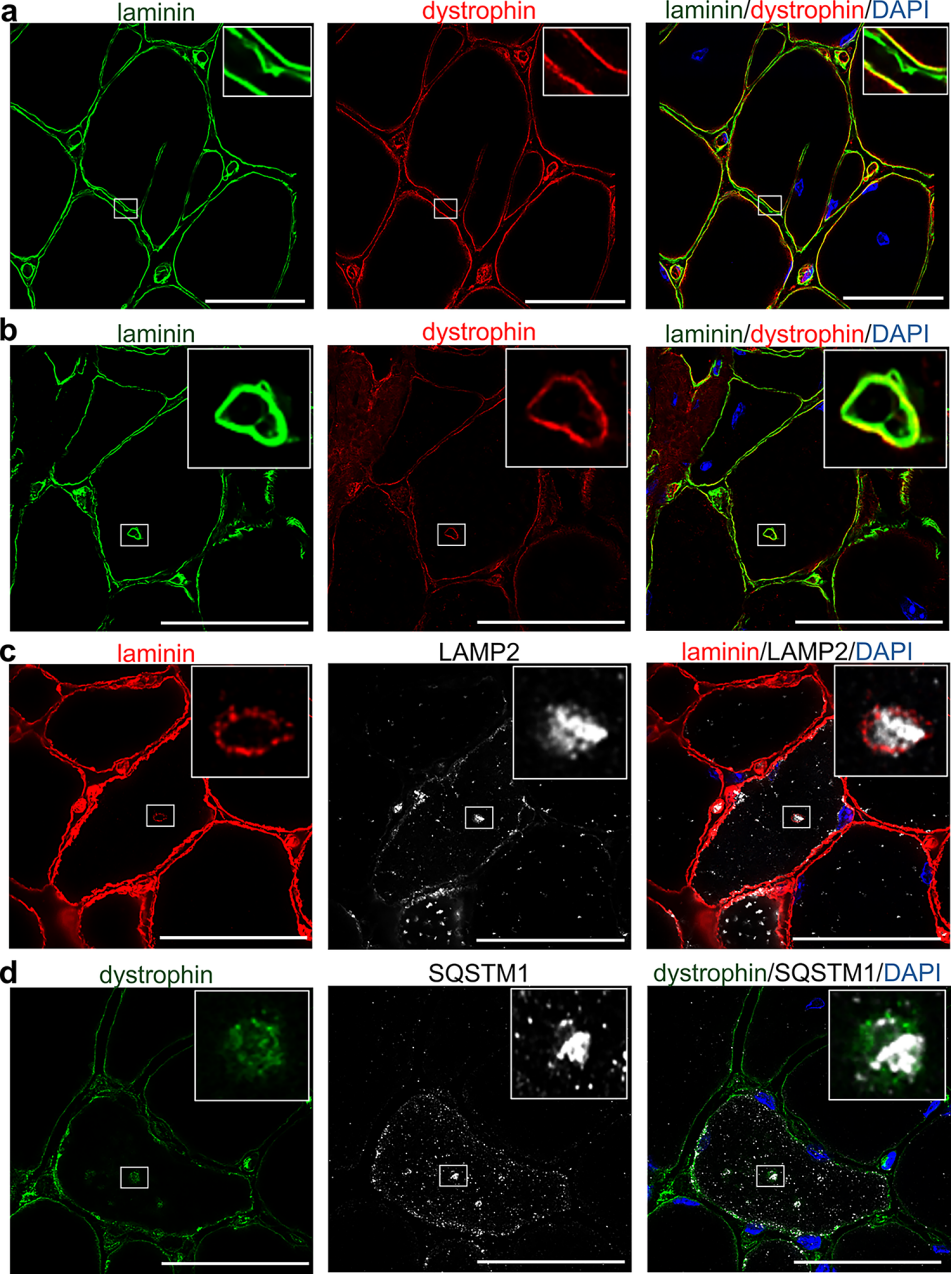
Skeletal muscle-specific deletion of *Vma21* results in basal lamina reduplication and autophagic vacuoles with sarcolemma features (AVSFs). Co-immunofluorescence on cryosections from gastrocnemius muscle of HSA-CreERT2;*Vma21*^fl/Y^ mice after 4 months on tamoxifen treatment using antibodies against laminin (green) and dystrophin (red) (**a**, **b**), laminin (red) and LAMP2 (white) (**c**), dystrophin (green) and SQSTM1 (white) (**d**). DAPI (blue) stains nuclei. Insets show higher-magnification views of the indicated regions. Scale bars: 50 μm

To determine whether skeletal muscle-specific deletion of *Vma21* recapitulates defining pathological features of X-linked myopathy with excessive autophagy (XMEA), we examined skeletal muscle sections for histopathological hallmarks of XMEA. Immunofluorescence staining for laminin and dystrophin revealed basal lamina reduplication in HSA-*Vma21*KO muscle fibers after 4 months of tamoxifen treatment (Fig. 7a). We also identified autophagic vacuoles with sarcolemmal features (AVSFs), marked by cytoplasmic vacuolar structures positive for both laminin and dystrophin (Fig. 7b). Additional enzyme histochemistry and immunofluorescence analyses further characterize these structures in skeletal muscle (Fig. S3). Further characterization showed that these vacuolar structures contained LAMP2 and SQSTM1 within their lumen (Figs.). Together, these findings demonstrate that skeletal muscle-specific deletion of *Vma21* recapitulates key histopathological hallmarks of XMEA, including basal lamina reduplication and AVSFs.

### Ultrastructural analysis reveals membrane-bound vacuolar structures in VMA21-deficient skeletal muscle

**Fig. 8:**
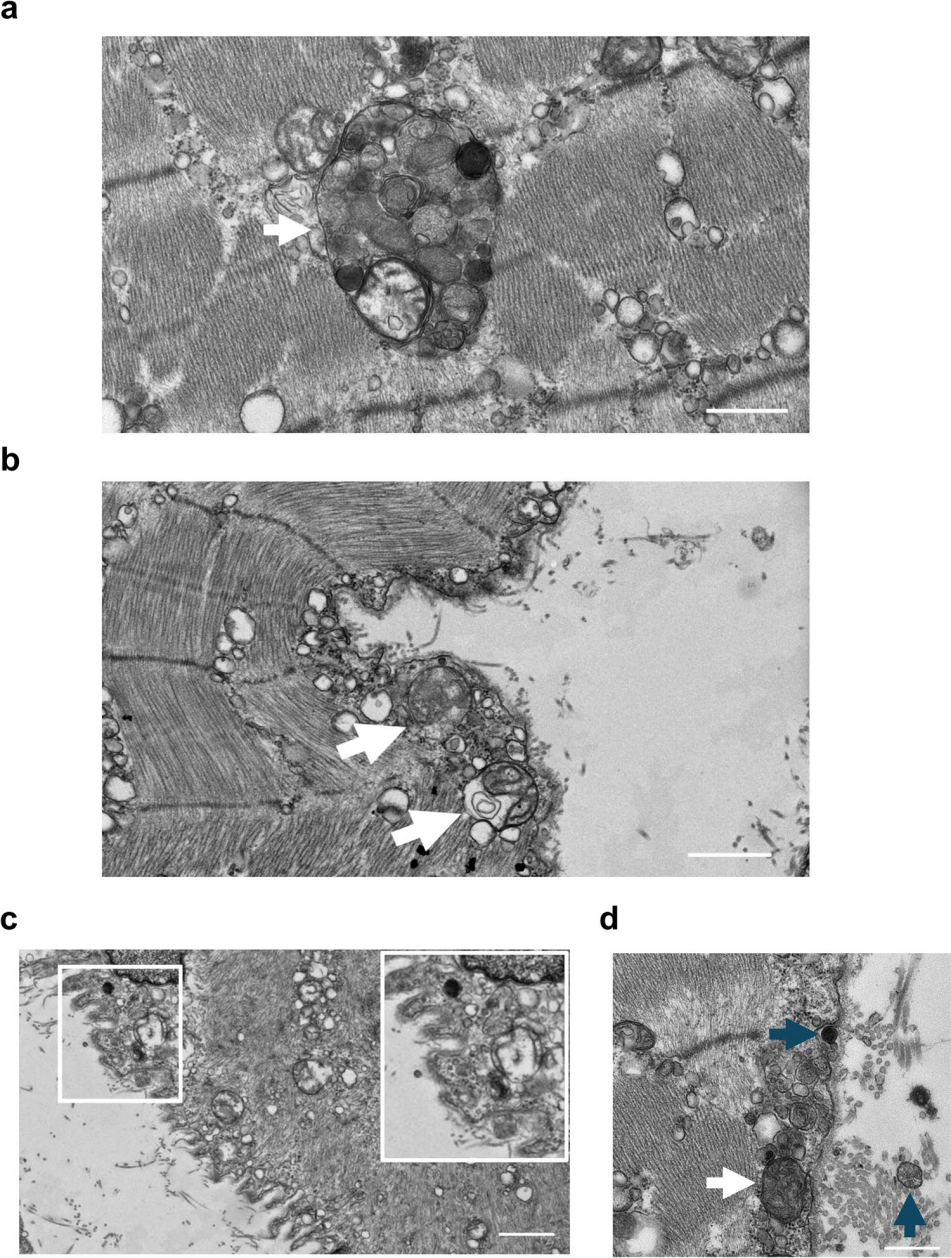
Skeletal muscle-specific deletion of *Vma21* leads to formation of vacuolar structures and membrane irregularity. Transmission electron microscopy of cryosections from gastrocnemius muscle of HSA-CreERT2;*Vma21*^fl/Y^ mice after 4 months on tamoxifen treatment showing a cytoplasmic vacuole containing electron-dense, undegraded material (**a**), accumulation of vacuolar structures at the sarcolemma (**b**), and regions of membrane irregularity (**c**). **d** Vesicle-like structures within the extracellular space adjacent to muscle fibers. White arrows indicate vacuolar structures. Blue arrows indicate extracellular vesicle-like structures. Inset shows a higher-magnification view of the indicated region. Scale bars: 600 nm (a); 800 nm (b); 1 μm (**c**); 500 nm (**d**)

Deletion of *Vma21* resulted in the presence of membrane-bound vacuoles containing partially degraded material within skeletal muscle fibers (Fig. 8a). Similar vacuolar structures were frequently observed accumulating in the subsarcolemmal region (Fig. 8b). In addition, regions of membrane irregularity associated with clusters of vesicular structures were observed along the fiber periphery (Fig. 8c). Vesicle-like structures were also observed within the extracellular space adjacent to muscle fibers (Fig. 8d). Together, these findings demonstrate the presence of membrane-associated vacuoles and vesicular structures in *Vma21*-deficient skeletal muscle.

### CD63 accumulation and colocalization with C5b-9 in VMA21-deficient skeletal muscle and human XMEA muscle

**Fig. 9:**
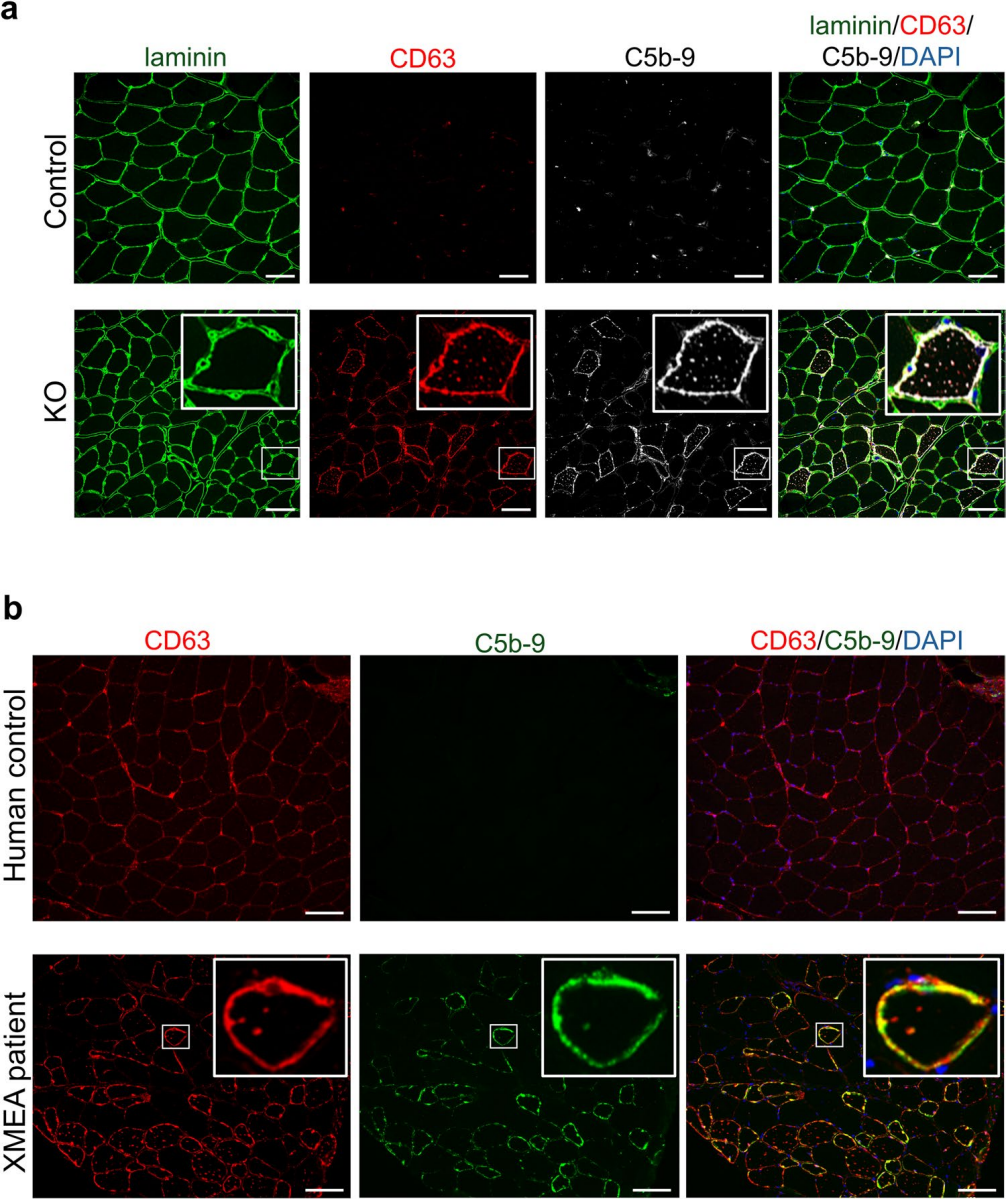
Vma21 deficiency is associated with CD63 accumulation and colocalization with C5b-9 in mouse skeletal muscle and human XMEA biopsies. **a** Co-immunofluorescence staining of gastrocnemius muscle cryosections from *Vma21*^fl/Y^ (control) or HSA-CreERT2;*Vma21*^fl/Y^ (KO) mice after 4 months of tamoxifen treatment using antibodies against laminin (green), CD63 (red), and C5b-9 (white). **b** Co-immunofluorescence staining of muscle biopsy sections from a control individual and an individual with XMEA using antibodies against CD63 (red) and C5b-9 (green). DAPI (blue) stains nuclei. Insets show higher-magnification views of the indicated regions. Scale bars: 50 μm (A); 100 μm (B)

Given the peripheral accumulation of vacuoles in Vma21-deficient muscle fibers, which has previously been hypothesized to be associated with increased exocytosis (10), we examined the distribution of the late endosomal/exosomal marker CD63. Immunofluorescence analysis of gastrocnemius muscle after four months of tamoxifen treatment revealed minimal CD63 staining in skeletal muscle from control littermates. In contrast, HSA-*Vma21*KO muscle fibers displayed a marked increase in CD63 positive structures, which were frequently enriched at the fiber periphery. Notably, CD63 staining robustly colocalized with the complement membrane attack complex C5b-9 at the sarcolemma of HSA-*Vma21*KO muscle fibers (Fig. 9a). Analysis of skeletal muscle biopsies from individuals with XMEA revealed a similar increase in CD63 staining and its colocalization with C5b-9 compared with control samples (Fig. 9b), indicating that this feature is conserved between the mouse model and human disease.

## Discussion

X-Linked myopathy with excessive autophagy (XMEA) is a rare genetic muscle disease caused by mutations in *Vma21,* yet the mechanisms linking Vma21 deficiency to progressive muscle pathology in vivo remain poorly understood. A major limitation in the field has been the lack of validated mammalian models that faithfully recapitulate the defining histopathological features of XMEA. In this study, we generated and characterized conditional *Vma21* knockout mouse models to establish an in vivo system that phenocopies core aspects of XMEA pathology, enables mechanistic investigation of disease progression, and provides a preclinical platform to evaluate potential therapeutic strategies.

Combined deletion of *Vma21* in skeletal and cardiac muscle in the MCK-Cre;*Vma21*^fl/Y^ strain resulted in rapid postnatal lethality driven by severe cardiomyopathy associated with accumulation of lysosomal and autophagy-related proteins, suggesting autophagic dysregulation. These findings are consistent with prior studies demonstrating that Vma21 is essential for autophagy function [6], and extend these observations by establishing that cardiac muscle is sensitive to Vma21 deficiency. A notable distinction between our MCK-Cre mouse model and the typical clinical presentation of XMEA is the severity of cardiomyopathy observed in mice, whereas cardiac involvement in patients is relatively uncommon [10, 11]. This discrepancy is most plausibly explained by differences in the degree of Vma21 loss. In individuals with XMEA, pathogenic variants reduce but do not completely abolish Vma21 transcript, consistent with a hypomorphic mechanism and residual Vma21 function [13]. In contrast, Cre-mediated recombination in the MCK-Cre model results in near-complete loss of Vma21 in both skeletal and cardiac muscle. Our findings suggest that cardiac muscle exhibits a lower tolerance for severe autophagic impairment than skeletal muscle under conditions of profound Vma21 deficiency. This interpretation is supported by the absence of evident skeletal muscle pathology at the time of death in MCK-Cre mice despite pronounced cardiomyopathy with autophagic pathology. Preferential cardiac involvement has also been described in other vacuolar myopathies associated with impaired autophagy. For example, Danon disease, caused by deficiency of the lysosomal membrane protein LAMP2, is characterized by severe cardiomyopathy together with skeletal muscle pathology [4, 5]. These observations suggest that cardiac muscle may be particularly sensitive to disruptions in lysosomal and autophagy-related pathways, providing a possible explanation for the pronounced cardiac phenotype observed in the MCK-Cre;*Vma21*^fl/Y^ mice.

In addition to differences in the degree of Vma21 loss, species-specific factors may also contribute to the contrast between the severe cardiomyopathy observed in the MCK-Cre mouse model and the predominantly skeletal muscle phenotype seen in individuals with XMEA. It is possible that human cardiomyocytes possess compensatory mechanisms that partially buffer the consequences of reduced Vma21 function, such as greater tolerance for impaired lysosomal acidification, increased engagement of alternative proteostasis pathways, or differences in lysosomal turnover and metabolic demand. Although speculative, these possibilities highlight the importance of considering both gene dosage and species-specific context when interpreting disease mechanisms. Importantly, emerging clinical reports describing cardiac abnormalities in a subset of XMEA patients indicate that the heart can be affected in this disorder, although cardiac involvement appears to be relatively uncommon [10, 11]. From a clinical perspective, our findings suggest that cardiac involvement in XMEA may be underrecognized and underscore the importance of systematic cardiac evaluation in affected individuals.

Because early cardiac failure precluded the development of overt skeletal muscle pathology in the MCK-Cre model, we generated a skeletal muscle-specific inducible *Vma21* knockout mouse strain. Using the HSA-CreERT2;*Vma21*^fl/Y^ model, we observed a progressive myopathy that mirrors key features of human XMEA. Following prolonged *Vma21* deletion, skeletal muscle developed increased centralized nuclei, fiber size variability, and fiber splitting. Consistent with observations from XMEA patient muscle biopsies [10], skeletal muscle-specific loss of VMA21 resulted in autophagic dysregulation, evidenced by accumulation of LAMP2, LC3B-II, and poly-ubiquitinated proteins and by enrichment of autophagy-related structures at the fiber periphery. Notably, transcript levels of *Lc3b* and *Sqstm1* were not significantly altered, indicating that protein accumulation reflects impaired autophagy rather than increased gene expression. The absence of increased SQSTM1 signal by immunoblotting despite robust accumulation detected by immunofluorescence may reflect sequestration of SQSTM1 within membrane-associated or detergent-insoluble compartments, limiting its recovery in total muscle lysates. The discordance in SQSTM1 accumulation between heart and skeletal muscle may reflect differences in disease severity and tissue heterogeneity.

An additional feature of this inducible skeletal muscle model is the delayed onset of pathology. After two months of tamoxifen treatment, despite efficient loss of Vma21, we did not detect evident myopathic changes or alterations in autophagy-associated proteins. At three months, however, skeletal muscle exhibited increased fiber size variability together with early features of myopathy, including internal nucleation and fiber splitting, suggestive of a transitional phase during disease progression. By four months of tamoxifen treatment, myopathic changes were clearly apparent and were accompanied by alterations in autophagy-associated proteins. These observations suggest that skeletal muscle fibers may initially tolerate reduced Vma21 function through compensatory mechanisms, with pathological changes emerging once the burden of Vma21 deficiency exceeds the capacity of these adaptive responses. One possible contributor to this delayed phenotype is the relatively long lifespan of lysosomes in mature muscle fibers. Lysosomes assembled prior to *Vma21* deletion may initially remain functional, whereas newly formed lysosomes lacking properly assembled V-ATPase complexes may gradually accumulate. Over time, this decline in lysosomal function may lead to the accumulation of autophagic material and the development of myofiber pathology.

Autophagic vacuoles with sarcolemmal features (AVSFs) and reduplication of the basal lamina are defining pathological hallmarks of X-linked myopathy with excessive autophagy (XMEA) [8, 10]. However, faithful recapitulation of these structures in experimental models has proven challenging. For example, a recently described zebrafish *Vma21* loss-of-function model exhibited autophagic impairment but did not develop AVSFs or basal lamina reduplication [14]. In contrast, our inducible skeletal muscle model demonstrates that sustained loss of Vma21 in mammalian muscle is sufficient to drive both AVSF formation and basal lamina reduplication, as evidenced by co-immunofluorescence of laminin and dystrophin. These vacuoles contained lysosomal and autophagy-associated markers within their lumen, consistent with their autophagic origin. Ultrastructural analysis further revealed membrane-bound cytoplasmic vacuoles containing partially degraded material, further supporting the presence of autophagic vacuolar pathology consistent with that observed in muscle biopsies from individuals with XMEA.

In addition to autophagic dysregulation, basal lamina reduplication, and AVSF formation, our data indicate that Vma21 deficiency is associated with altered vesicle organization at the muscle fiber periphery. Ultrastructural analysis revealed membrane-bound vacuoles containing partially degraded material together with clusters of vesicular structures accumulating in the subsarcolemmal region. Vesicle-like structures were also observed within the extracellular space adjacent to muscle fibers. Consistent with these observations, we detected increased CD63-positive structures within mutant muscle fibers, frequently enriched at the fiber periphery. A similar increase in CD63 staining was observed in skeletal muscle biopsies from patients with XMEA, indicating that this feature is conserved between the mouse model and human disease. The peripheral accumulation of vacuoles in XMEA muscle has previously been hypothesized to reflect increased exocytosis of undegraded material, and the enrichment of CD63 at the sarcolemma is consistent with altered vesicle trafficking in this region.

Notably, CD63-positive structures robustly colocalized with the complement membrane attack complex C5b-9 at the sarcolemma in both Vma21-deficient mouse muscle and human XMEA biopsies. Complement deposition has previously been reported in XMEA muscle [8, 10], although its origin and significance have remained unclear. Our observations raise the possibility that altered vesicle trafficking or increased exposure of vesicle-associated components may contribute to complement deposition at the muscle fiber surface. Although the functional consequences of this association remain to be determined, these findings suggest a link between autophagic dysregulation, altered vesicle trafficking, and complement deposition in XMEA. Together, these results support a model in which Vma21 deficiency leads to autophagic dysregulation and altered vesicle trafficking that promote membrane remodeling and complement deposition at the sarcolemma, potentially contributing to skeletal muscle pathology in XMEA.

The prominent sarcolemmal deposition of C5b-9 also raises the possibility that complement inhibition may represent a potential therapeutic strategy in XMEA. Consistent with this idea, treatment with the C5 inhibitor eculizumab has recently been reported to improve clinical measures in a patient with dysferlinopathy [15], another muscle disease characterized by complement deposition at the sarcolemma. Although the mechanisms driving complement activation may differ between these disorders, these findings support the therapeutic potential of complement inhibition and suggest that complement-directed therapies could be evaluated in Vma21-deficient mouse models to determine whether complement contributes to disease progression in XMEA.

Several limitations of this study should be acknowledged. The inducible knockout model represents near-complete loss of Vma21, whereas most patients retain partial protein function. In addition, the temporal relationship between autophagic dysregulation, altered vesicle trafficking, membrane remodeling, and muscle weakness remains to be fully defined. Only male mice were analyzed in this study. Given the X-linked nature of XMEA, future studies including female mice will be important to determine whether similar pathological features develop and to better understand potential sex-specific differences in disease severity. Finally, additional studies will be required to determine whether altered vesicle trafficking contributes directly to disease progression or represents a compensatory response to impaired autophagy.

In summary, our study establishes conditional mouse models that recapitulate the defining pathological features of XMEA, including progressive skeletal muscle myopathy, autophagic dysregulation, AVSF formation, basal lamina reduplication, and accumulation of vacuolar structures at the sarcolemma. Importantly, these findings provide evidence linking Vma21 deficiency to increased CD63-positive structures and their colocalization with the complement membrane attack complex C5b-9 in skeletal muscle, a feature that is conserved in human XMEA biopsies. Together, these models provide a robust mammalian platform for dissecting disease mechanisms and for evaluating therapeutic strategies aimed at restoring autophagy and vesicle trafficking in XMEA.

## Supplementary Information

Below is the link to the electronic supplementary material.

Supplementary file1 Fig. S1. Bulk autophagy markers are unchanged in skeletal muscle of MCK-Cre/Vma21fl/Y mice at P20. Immunoblot analysis of LC3B and SQSTM1 in tibialis anterior (TA; a), gastrocnemius (Gast; b), and quadriceps (Quad; c) muscles from Vma21fl/Y (Control) and MCK-Cre;Vma21fl/Y (KO) mice at postnatal day 20 (P20). GAPDH was used as a loading control. Comparison between groups was performed using an unpaired two-tailed Student’s t-test. *p < 0.05; **p < 0.01; ***p < 0.001; n.s., not significant. For gastrocnemius and quadriceps samples, LC3B and SQSTM1 immunoblots were obtained from the same membranes and share the same GAPDH loading control. Tibialis anterior samples were run on separate membranes and therefore have independent GAPDH controls. (TIF 2991 KB)

Supplementary file2 Fig. S2 Bulk autophagy markers are unchanged in skeletal muscle of HSA-CreERT2;Vma21fl/Y mice after two months of tamoxifen treatment. Co-immunofluorescence on cryosections from gastrocnemius muscle of Vma21fl/Y (Control) or HSA-CreERT2;Vma21fl/Y (KO) mice after 2 months of tamoxifen treatment staining for dystrophin (green) and LAMP2 (white) (a), caveolin 3 (green) and poly-ubiquitinated proteins (P4D1) (white) (b), dystrophin (green) and LC3B (white) (c), or dystrophin (green) and SQSTM1 (white) (d). DAPI (blue) stains nuclei. Scale bars: 50 μm. (TIF 19261 KB)

Supplementary file3 Fig. S3 Enzyme histochemistry and immunofluorescence of skeletal muscle from Vma21fl/Y (Control) and HSA-CreERT2;Vma21fl/Y (KO) mice. (a) Esterase enzyme histochemistry on quadriceps skeletal muscle sections from Control and KO mice after 6 months of tamoxifen treatment. (b) Acid phosphatase enzyme histochemistry on quadriceps skeletal muscle sections from Control and KO mice after 6 months of tamoxifen treatment. (c) Immunofluorescence staining of gastrocnemius skeletal muscle sections from KO mice after 4 months of tamoxifen treatment using antibodies against laminin or dystrophin. Scale bars: 50 μm. (TIF 18099 KB)

## Data Availability

The data that support the findings of this study are available from the corresponding author upon reasonable request.
