# Supplementary figures and images for: VMA21 deficiency leads to autophagic dysregulation and altered vesicle trafficking in X-linked myopathy with excessive autophagy
Source: Acta Neuropathol. 2026 Jun 26;151(1):73. doi: 10.1007/s00401-026-03044-z (PMC13309440; doi:10.1007/s00401-026-03044-z)

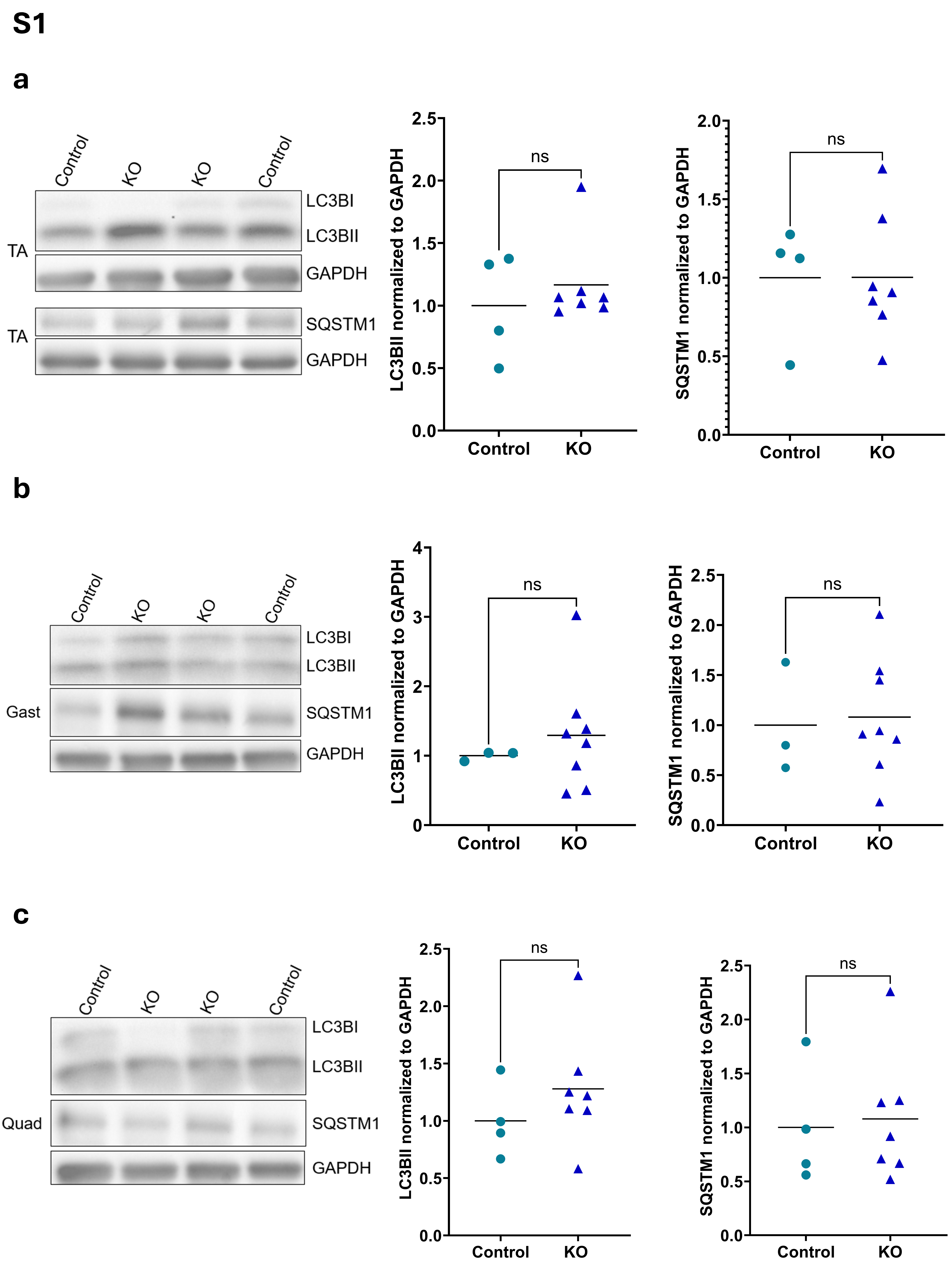

Supplement: Supplementary file 1 — Supplementary file1 Fig. S1. Bulk autophagy markers are unchanged in skeletal muscle of MCK-Cre/Vma21fl/Y mice at P20. Immunoblot analysis of LC3B and SQSTM1 in tibialis anterior (TA; a), gastrocnemius (Gast; b), and quadriceps (Quad; c) muscles from Vma21fl/Y (Control) and MCK-Cre;Vma21fl/Y (KO) mice at postnatal day 20 (P20). GAPDH was used as a loading control. Comparison between groups was performed using an unpaired two-tailed Student’s t-test. *p < 0.05; **p < 0.01; ***p < 0.001; n.s., not significant. For gastrocnemius and quadriceps samples, LC3B and SQSTM1 immunoblots were obtained from the same membranes and share the same GAPDH loading control. Tibialis anterior samples were run on separate membranes and therefore have independent GAPDH controls. (TIF 2991 KB) [file 401_2026_3044_MOESM1_ESM.tif]

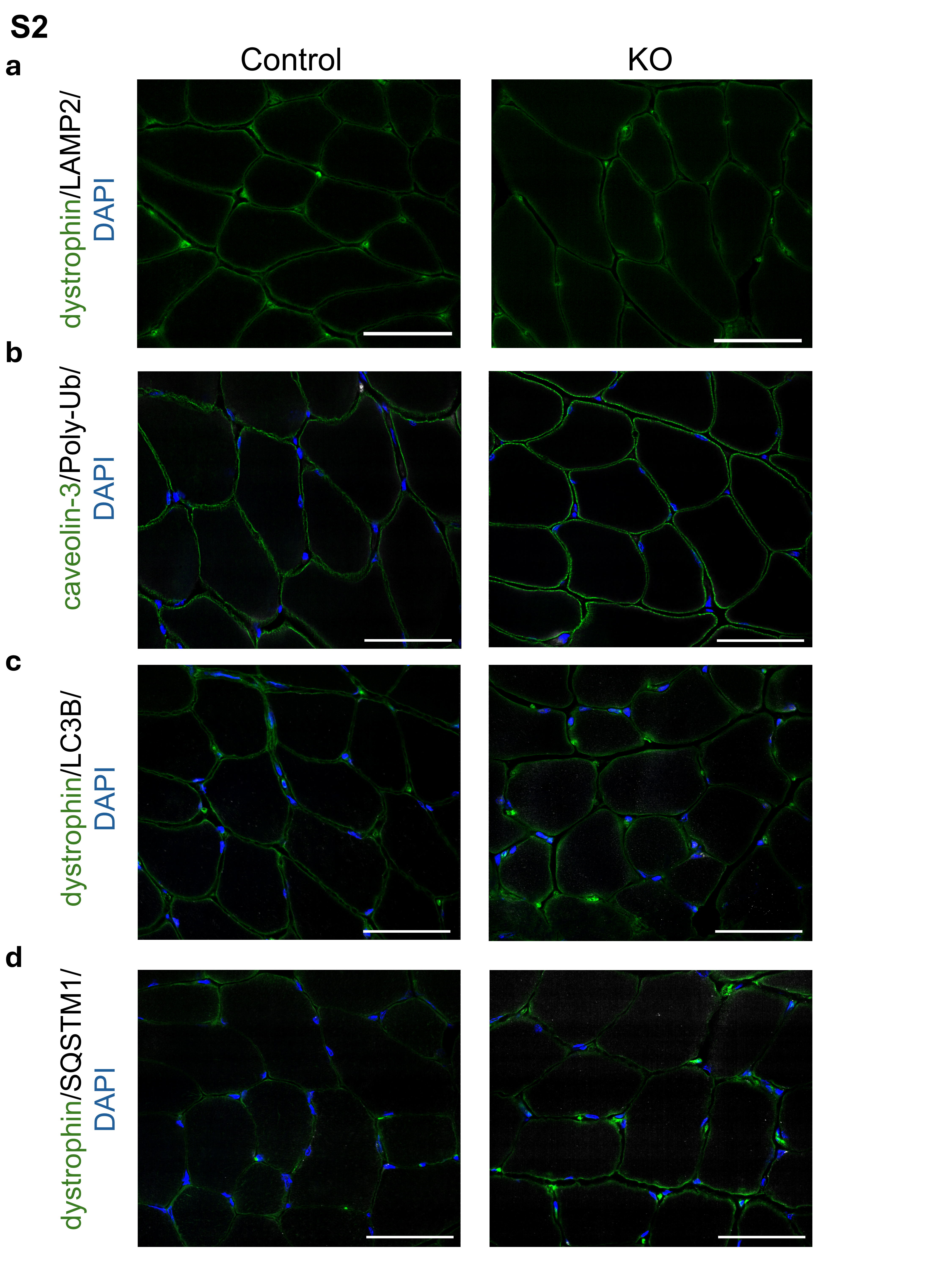

Supplement: Supplementary file 2 — Supplementary file2 Fig. S2 Bulk autophagy markers are unchanged in skeletal muscle of HSA-CreERT2;Vma21fl/Y mice after two months of tamoxifen treatment. Co-immunofluorescence on cryosections from gastrocnemius muscle of Vma21fl/Y (Control) or HSA-CreERT2;Vma21fl/Y (KO) mice after 2 months of tamoxifen treatment staining for dystrophin (green) and LAMP2 (white) (a), caveolin 3 (green) and poly-ubiquitinated proteins (P4D1) (white) (b), dystrophin (green) and LC3B (white) (c), or dystrophin (green) and SQSTM1 (white) (d). DAPI (blue) stains nuclei. Scale bars: 50 μm. (TIF 19261 KB) [file 401_2026_3044_MOESM2_ESM.tif]

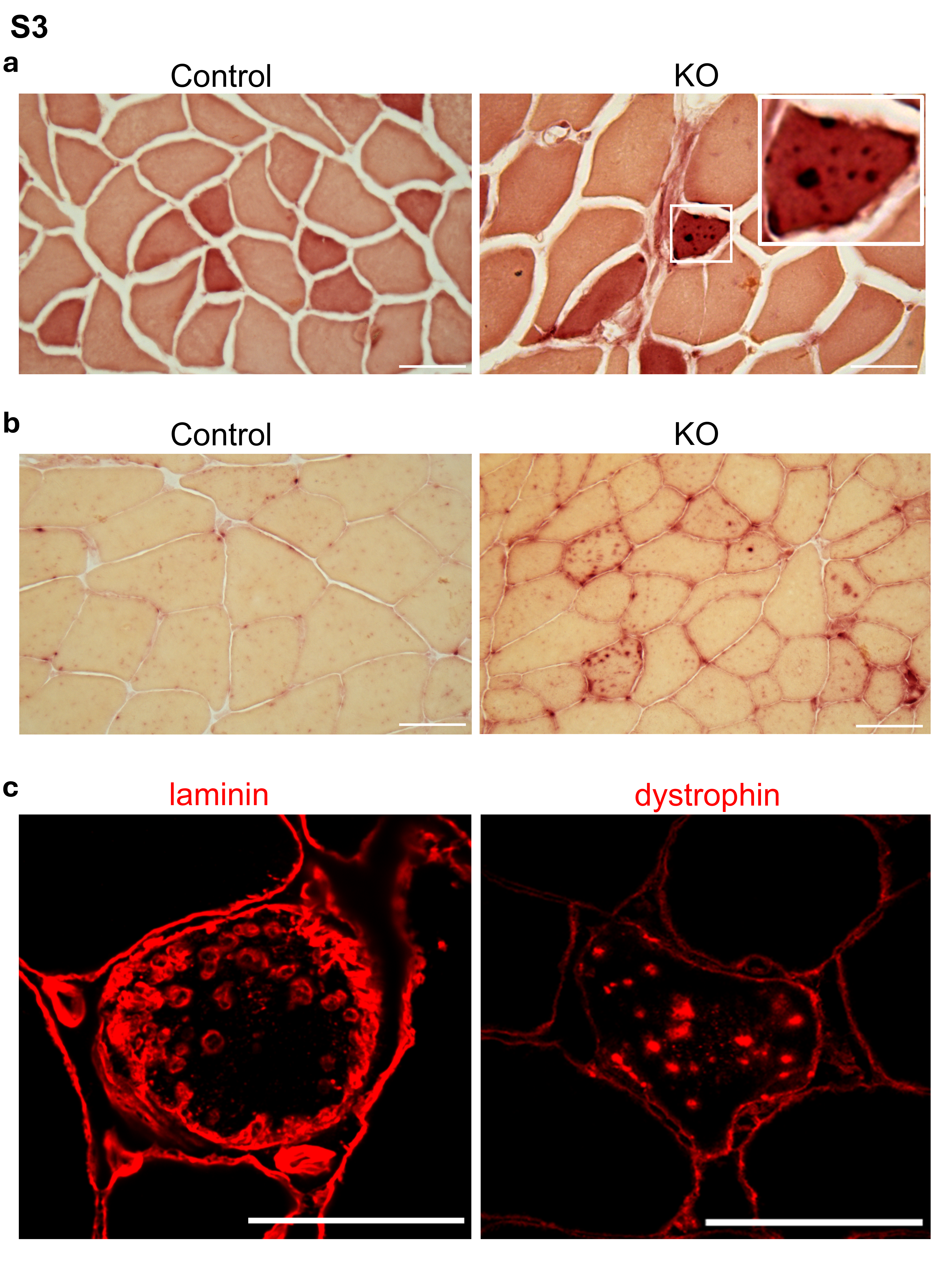

Supplement: Supplementary file 3 — Supplementary file3 Fig. S3 Enzyme histochemistry and immunofluorescence of skeletal muscle from Vma21fl/Y (Control) and HSA-CreERT2;Vma21fl/Y (KO) mice. (a) Esterase enzyme histochemistry on quadriceps skeletal muscle sections from Control and KO mice after 6 months of tamoxifen treatment. (b) Acid phosphatase enzyme histochemistry on quadriceps skeletal muscle sections from Control and KO mice after 6 months of tamoxifen treatment. (c) Immunofluorescence staining of gastrocnemius skeletal muscle sections from KO mice after 4 months of tamoxifen treatment using antibodies against laminin or dystrophin. Scale bars: 50 μm. (TIF 18099 KB) [file 401_2026_3044_MOESM3_ESM.tif]
